# Neoadjuvant Treatment in Muscle-Invasive Bladder Cancer: From the Beginning to the Latest Developments

**DOI:** 10.3389/fonc.2022.912699

**Published:** 2022-07-22

**Authors:** Giandomenico Roviello, Martina Catalano, Raffaella Santi, Matteo Santoni, Ilaria Camilla Galli, Andrea Amorosi, Wojciech Polom, Ugo De Giorgi, Gabriella Nesi

**Affiliations:** ^1^ Department of Health Sciences, University of Florence, Florence, Italy; ^2^ School of Human Health Sciences, University of Florence, Florence, Italy; ^3^ Oncology Unit, Macerata Hospital, Macerata, Italy; ^4^ Histopathology and Molecular Diagnostics, Careggi Teaching Hospital, Florence, Italy; ^5^ Department of Health Sciences, University of Catanzaro, Catanzaro, Italy; ^6^ Department of Urology, Faculty of Medicine, Medical University of Gdansk, Gdansk, Poland; ^7^ Department of Medical Oncology, IRCCS Istituto Romagnolo per lo Studio dei Tumori (IRST) Dino Amadori, Meldola, Italy

**Keywords:** muscle-invasive bladder cancer, neoadjuvant chemotherapy, immunotherapy, combined therapy, biomarkers, molecular subtypes

## Abstract

Urothelial carcinoma of the bladder is one of the most prevalent cancers worldwide, diagnosed as muscle invasive in 25% of cases. Although several studies have demonstrated an overall 5% absolute survival benefit at 5 years with cisplatin-based combination neoadjuvant treatment, administration of chemotherapy prior to radical cystectomy (RC) in muscle-invasive bladder cancer (MIBC) patients is still a matter of debate. This may be due to the perceived modest survival benefit, cisplatin-based chemotherapy ineligibility, or fear of delaying potentially curative surgery in non-responders. However, immunotherapy and novel targeted therapies have shown to prolong survival in advanced disease and are under investigation in the neoadjuvant and adjuvant settings to reduce systemic relapse and improve cure rates. Genomic characterization of MIBC could help select the most effective chemotherapeutic regimen for the individual patient. Large cohort studies on neoadjuvant treatments with immune checkpoint inhibitors (ICIs) and molecular therapies, alone or combined with chemotherapy, are ongoing. In this review, we trace the development of neoadjuvant therapy in MIBC and explore recent advances that may soon change clinical practice.

## Introduction

Bladder cancer (BC) accounts for almost 600,000 new cases and over 200,000 deaths worldwide (1). Muscle-invasive bladder cancer (MIBC) constitutes 25% of newly diagnosed BC cases (2), and in approximately 50% of these patients treated with radical cystectomy (RC), the disease recurs within two years (3). To date, cisplatin-based neoadjuvant chemotherapy (NAC) is the standard of care for MIBC and is associated with a 5% absolute survival benefit at 5 years and a 14% relative risk reduction for death (4). Chemotherapy prior to RC has long been a matter of debate. Although administration of NAC for MIBC has increased over the years, it still does not meet actual needs (5), particularly in cT2 BC for which it is currently recommended in clinical guidelines (6, 7). Multidisciplinary management is of paramount importance in this disease setting. Indeed, with the development of new cytotoxic and targeted therapies, and specifically immune checkpoint inhibitors (ICIs), large ongoing prospective studies have been designed to test their efficacy either alone or in combination in the neoadjuvant setting. Furthermore, identification of biomarkers, such as molecular phenotype and DNA damage repair, appears to predict response to cisplatin-based NAC. In this article, we review data in support of chemotherapy, molecular therapy and immunotherapy in early-stage MIBC, and discuss the impact of molecular biology in clinical practice.

## Methods

From October 2021 to February 2022, we searched PubMed database for studies containing the keywords “neoadjuvant chemotherapy”, “muscle-invasive bladder cancer”, “neoadjuvant immunotherapy”, “biomarkers of response”, and “neoadjuvant combination therapy”. Several results were analyzed for review; all studies involved MIBC patients who were candidates for surgery upfront or after neoadjuvant therapy. We also searched the clinical trial.gov database for all phase II/III “active” or “active, not recruiting” studies on neoadjuvant therapy for MIBC.

## Neoadjuvant Chemotherapy in MIBC

Cisplatin-based NAC is the treatment recommended by the National Comprehensive Cancer Network (NCCN) and the European Association of Urology (EAU) for patients with MIBC (cT2-4a or positive lymph nodes, N1) and fit for cisplatin (6, 7). Compared with RC alone, neoadjuvant cisplatin-based combination chemotherapy has improved overall survival (OS) and lowered the risk of recurrence. The clinical benefits of NAC in MIBC have been highlighted by several randomized phase III studies, although the ideal NAC regimen has not yet been established (8–10). Cisplatin-based NAC was first tested in the 1980s as a potential treatment strategy for MIBC. NAC based on methotrexate, vinblastine, doxorubicin and cisplatin (MVAC) was administered to 30 MIBC patients treated with RC, achieving a 33% pathologic complete response (pCR) and 17% disease downstaging to <pT2N0 (11).

A combined analysis of two separate trials with similar patient populations showed an 8% improvement in the 5-year OS rate with NAC (56%) compared with the control group (48%), and a 20% reduction in the relative probability of death (9). Regarding local radical treatment alone versus neoadjuvant cisplatin, methotrexate and vinblastine (CMV), an international multicenter study (BA06 30894 trial) demonstrated on first analysis a non-significant 15% reduction in the risk of death with neoadjuvant CMV (8). Updated results revealed a statistically significant 16% reduction in the risk of death and a 6% increase in the 10-year survival rate with neoadjuvant CMV compared to the control group (12). Further meta-analyses assessing the clinical benefits of NAC confirmed a 5% improvement of OS in MIBC (13–15).

In the SWOG trial, MVAC-based NAC was tested against surgery alone in 317 patients with cT2-4aN0M0 BC. Median OS was 77 months in the NAC group and 46 months in the surgery alone group, while 5-year survival rate was 57% and 43%, respectively (10).

Two small, single-arm phase II trials investigated a modified MVAC regimen consisting of dose dense MVAC (dd-MVAC) with granulocyte colony-stimulating factor (G-CSF) support, evaluating NAC efficacy and safety in cT2-4N0 BC (16, 17). Of 39 patients, 49% achieved pathologic response, defined as downstaging to ≤pT1N0M0, with 10% showing grade 3 or higher treatment-related toxicities (16). Likewise, 38% pCR (pT0) rates and 52% downstaging to non-muscle-invasive disease (NMIBC) were observed by Plimack et al., with the majority of patients (82%) experiencing only grade 1–2 treatment-related toxicities (17).

The combination of gemcitabine and cisplatin (GC) is another regimen utilized in the neoadjuvant setting, showing similar OS, progression-free survival (PFS), and downstaging to pT0/pT1, but lower toxicity when compared to conventional MVAC (18–23). A randomized phase III trial assessed the efficacy of neoadjuvant treatment with GC and dd-MVAC in 537 patients (24). Overall, pCR was seen in 36% and 42% of GC and dd-MVAC patients (p=0.2), while downstaging to organ-confined disease (<ypT3pN0) was achieved in 63% and 77% (p=0.001), respectively. Grade 3 or higher hematologic toxicities were similar, 55% in the GC group and 52% in the dd-MVAC group. Contrariwise, grade 3 or higher gastrointestinal toxicities (p=0.003) and asthenia (p<0.001) were more frequent in the dd-MVAC arm. Results of NAC trials in MIBC are summarized in **Table 1**.

**Table 1:** Main clinical trials of neoadjuvant cisplatin-based chemotherapy for MIBC.

**Trial (ref.)**	**Phase**	**N of patients**	**Regimen**	**Duration of NAC, weeks**	**pCR (pT0N0) rates, %**	**Downstaging (<pT2), %**
**SWOG-8710** (10)	III	317	MVAC	14	38	44
**BA0630894** (8)	III	976	CMV	NA	NA	NA
**Choueiri et al.** (16)	II	651	dd-MVAC	8	26	49
**Plimack et al.** (17)	II	44	dd-MVAC	6	38	53
**Iyer et al.** (18)	Retrospective	154	GC	12	21	46
**Dash et al.** (20)	Retrospective	42	GC	12	26	36

Neoadjuvant chemotherapy (NAC), muscle-invasive Bladder cancer (MIBC), gemcitabine-cisplatin (GC), number (N), pathologic complete response (pCR), dose dense methotrexate-vinblastine-doxorubicin-cisplatin (dd-MVAC), cisplatin, methotrexate, and vinblastine (CMV).

### Cystectomy and Lymphadenectomy in Patients Treated With Neoadjuvant Therapy

Surgery is the standard approach for patients with MIBC or refractory NMIBC. Selection of MIBC patients as candidates for NAC requires careful consideration. It has recently emerged that impaired nutritional status due to neoadjuvant therapy is a key factor. In a study led by Cohen et al., variations in nutritional status were assessed by changes in smooth muscle index (SMI), calculated through cross-sectional imaging of psoas muscle area (25). These authors reported that SMI decline after neoadjuvant therapy was significantly associated with the risk of post-RC complications, including ileus and infections.

With the intent of determining the outcome of patients subjected to RC following NAC, Mir et al. developed and internally validated a nomogram predicting BC-specific mortality (BCSM) in MIBC patients (26). At multivariate analysis, lymph node metastasis (hazard ratio [HR] 1.90, 95% CI: 1.4-2.6), positive surgical margins (HR 2.01, 95% CI: 1.3-2.9) and pathologic stage ypT3-4 (HR 5.9, 95% CI: 3.8-9.3) were correlated with reduced BCSM, thus suggesting the potential use of this nomogram to identify patients eligible for adjuvant approaches or personalized follow-up.

Pre-surgical evaluation through [18F] Fluoro-Deoxy-Glucose Positron Emission Tomography (FDG-PET) is reserved for patients with suspected lymph node involvement at computed tomography (CT) scan. In patients receiving neoadjuvant anti-programmed cell death-1 (PD-1) immunotherapy by pembrolizumab, the sensitivity and specificity of PET/CT to predict lymph node metastasis was investigated before and after treatment (27). In this study, 4 of 7 patients (57%) with baseline FDG-uptake showed pathologic lymph node involvement versus 11 of 101 (11%) with no baseline FDG-uptake. Six of the 7 patients responded to neoadjuvant pembrolizumab, implying the necessity to further investigate and validate the use of PET/CT to determine those MIBC patients who are better candidates for neoadjuvant immunotherapy. Briganti et al. were the first to demonstrate the surgical safety of RC and pelvic lymph node dissection (PLND) in non-metastatic MIBC patients receiving neoadjuvant therapy with checkpoint inhibitors (28). They found that 77% and 34% of patients experienced any-grade and high-grade complications, respectively. The most frequent complications were fever (52%) and ileus (31%), with no perioperative mortality cases observed at 90 days.

According to the EAU guidelines, the high specificity of DWI-MRI seems to accurately predict pCR and allow better patient selection for bladder-sparing protocols (7). Pre-operative MRI in different settings may therefore provide useful information regarding treatment response.

### Predictive Biomarkers of Response in Cisplatin-Based Chemotherapy

Cisplatin-based chemotherapy remains the standard treatment for advanced disease and perioperative (neoadjuvant) treatment of BC (29). Cisplatin crosslinks DNA in different ways, mainly forming adducts that prevent cell replication and induce cell death. DNA damage can manifest as single-strand breaks (SSBs), double-strand breaks (DSBs) or interstrand-crosslinks (30). Cancer cells rely on various mechanisms to repair DNA damage: excision repair, mismatch repair (MMR) or nucleotide excision repair (NER) for SSBs, while non-homologous end joining or homologous recombination (HR) can correct DSBs.

There are several reports on the genes involved in DNA damage repair (DDR) pathways, highlighting their predictive role as biomarkers of response to cisplatin (31) (**Table 2**). A panel of 34 DDR genes was analyzed in a study enrolling 100 advanced BC patients treated with platinum-based chemotherapy. Overall, 47 patients had at least one alteration, and median OS was significantly higher in these patients than in those without (23.7 vs. 13.0 months, p=0.006). A recent phase II trial, investigating a panel of 29 DDR genes in 49 patients administered neoadjuvant dose-dense GC, showed a greater response to chemotherapy, with a positive predictive value of 89% and a 2-year relapse-free survival of 100%, in patients with deleterious mutations (39).

**Table 2:** Association between biomarkers and response to NAC in MIBC.

**Biomarker**	**ERCC2 mutation**	**ERCC2 mutation**	**ATM/RB1/FANCC mutations**	**ERBB2 mutations**	**DDR gene alterations**	**High expression ERCC1**	**BRCA1 mutation**
**Number of patients**	50	48+54	34	71	34	57	57
**Response to cisplatin-based NAC**	Increased pathologic response	Improved OS	Improved pT<2 response and OS	Increased pT0 response	Increased pT0/pTis response	Association with worse prognosis	Negative correlation with pCR and OS
**Reference**	(32)	(33, 34)	(33)	(35)	(36)	(37)	(38)

Neoadjuvant chemotherapy (NAC), muscle-invasive bladder cancer (MIBC), excision repair (ERCC), breast cancer gene (BRCA), ATM serine/threonine kinase (ATM), RB transcriptional corepressor 1 (RB1), FA complementation group C (FANCC).

Excision repair 1 and 2 (ERCC1 and ERCC2) proteins, belonging to the NER pathway, have been correlated with cisplatin-based chemotherapy response. High ERCC1 expression has been associated with gain of NER pathway function that leads to increased DNA repair capacity and platinum resistance (40, 41). In preclinical studies, ERCC2 mutations have been linked to loss of NER pathway function that confers sensitivity to cisplatin and carboplatin, but not to doxorubicin and ionizing radiation or poly (ADP-ribose) polymerase (PARP) inhibitors (42). Van Allen et al. detected ERCC2 mutations in 36% of patients who responded effectively to chemotherapy (<ypT1) but not in non-responders (>ypT2) (32). Further studies reported ERCC2 mutations in 38% (17/45) of responders and in only 6% (3/53) of non-responders (30). Recently, ERCC2 mutations were observed more frequently in primary than in secondary MIBC (12% vs. 1.2%), and patients with primary MIBC attained higher pathologic response rates following NAC (42).

Efficacy of NAC in MIBC has also been related to mutations in the ATM serine/threonine kinase (ATM), RB transcriptional corepressor 1 (RB1) and FA complementation group C (FANCC) repair genes. Plimack et al. detected genomic alterations in these genes in 13 of 15 cisplatin-responders (87%), while none of the non-responders harbored these mutations (33). A recent update of this study revealed a statistically significant improved 5-year disease-specific survival in carriers of at least one mutation compared to patients with no mutation (90% vs. 49%, p=0.0015) (43). The phase II RETAIN trial is currently evaluating bladder preservation in patients with ATM, RB1, FANCC or ERCC2 mutations who have achieved complete response with NAC (44). The presence of DDR genomic alterations could well identify those patients likely to respond to NAC and benefit from a bladder-sparing approach.

Breast cancer type 1 and 2 (BRCA1 and BRCA2) are among frequently mutated homologous recombination (HR) genes in urothelial carcinoma (45). According to Font et al., increased BRCA1 mRNA expression is negatively associated with pathologic response and survival in MIBC patients receiving NAC (38).

Current evidence indicates that alterations in DNA repair pathways can provide prognostic and predictive information in cisplatin-treated BC patients. Prospective studies including a large number of patients are needed to confirm these findings, which could pave the way for novel treatments, such as PARP inhibitors in HR-deficient cancers (46).

## Neoadjuvant Immunotherapy in MIBC

Lately, immunotherapy has become an integral part of advanced and metastatic BC treatment (47–56). Between 2016 and 2017, monoclonal antibodies against the negative immunoregulatory human cell surface receptor PD-1 (nivolumab and pembrolizumab), and its ligand programmed death ligan 1 (PD-L1) (atezolizumab, avelumab and durvalumab) have been approved for metastatic urothelial cancer by the United States Food and Drug Administration (FDA). Owing to their clinical benefits in the metastatic setting, several ICIs are being investigated in neoadjuvant (**Table 3**) and adjuvant settings (65).

**Table 3:** Main clinical trials of neoadjuvant immunotherapy for MIBC.

**Trial (ref.)**	**Phase**	**N of patients**	**Regimen**	**Cycles of NAC**	**pCR (pT0N0) rates, %**	**Downstaging (<pT2), %**
**PURE-01** (57, 58)	II	114	Pembrolizumab	3	39	56
**ABACUS** (59)	II	95	Atezolizumab	2	31	39
**NABUCCO** (60)	I	24	Ipilimumab/nivolumab	2	46	58
**DUTRENEO** (61)	II	61	Durvalumab/tremelimumab	3	35	57
**BLASST-1** (62)	II	41	Nivolumab + GC	4	49	66
**HCRN GU14-188** (63, 64)	Ib/II	12/70	Pem + GC (cohort 1) Pem + Gem (cohort 2)	4	44 54	61 52

Neoadjuvant chemotherapy (NAC), number (N), muscle-invasive bladder cancer (MIBC), gemcitabine-cisplatin (GC), pathologic complete response (pCR), pembrolizumab (Pem), gemcitabine (Gem).

### ICIs as Single Agents

In two single-arm phase II trials, pembrolizumab and atezolizumab have been tested in the neoadjuvant setting. The PURE-01 trial assessed the activity of pembrolizumab (200 mg every 3 weeks) for three cycles as neoadjuvant treatment before RC in patients with cT2-3bN0 MIBC and predominant urothelial cancer histology (57). Of these patients, 92% were eligible for cisplatin. Neoadjuvant pembrolizumab yielded 42% pCR and 54% downstaging to NMIBC. In addition, pCR was recorded in 54.3% of patients with PD-L1 combined positive score (CPS) ≥10 and in 13.3% of patients with PD-L1 CPS <10. High-grade complications, defined according to the Clavien-Dindo classification, were observed in 34% of patients, with no perioperative mortality at 90 days (7). Pembrolizumab response was maintained after cystectomy in most patients, with 1- and 2-year event-free survival (EFS) rates of 84.5% and 71.7%, respectively (58). A statistically significant longer EFS was found in patients with complete response and high PD-L1 CPS.

The ABACUS trial investigated the efficacy and safety of two cycles of neoadjuvant atezolizumab (1200 mg every 3 weeks) prior to RC for MIBC (59). Contrary to the PURE-01 trial, all patients were ineligible for or refused cisplatin-based NAC. The rates of pCR and downstaging to NMIBC were 31% and 39%, respectively. Treatment-related grade 3-4 toxicities occurred in 12% of patients, and grade 3 or 4 surgical complications in 31% of cases.

### ICIs as Combination Therapy

ICI combination has proved promising in different settings and types of cancer (66). Indeed, combined anti-PD-1 and anti-cytotoxic T-lymphocyte antigen 4 (CTLA-4) blockade prompts complementary mechanisms of therapeutic checkpoint inhibition, leading to greater antitumor activity than *via* a single pathway (67–69).

In the NABUCCO study, 24 patients with stage III urothelial cancer were administered 3 mg/kg ipilimumab (day 1), 1 mg/kg nivolumab plus 3 mg/kg ipilimumab (day 22), and 3 mg/kg nivolumab (day 43) in the neoadjuvant setting (60). The primary endpoint was feasibility to resect within 12 weeks from start of treatment. A total of 23 (96%) patients underwent surgery within 12 weeks, and grade 3-4 immune-related adverse events (iAEs) manifested in 55% of cases. Furthermore, 46% of patients showed pCR, and 58% had no remaining invasive disease (pCR or pTisN0/pTaN0).

Another randomized phase II trial (DUTRENEO) compared neoadjuvant durvalumab plus tremelimumab versus chemotherapy in cisplatin-eligible patients with cT2-4aN0-1 BC, classified as immunologically “hot” or “cold” according to the tumor immune score devised by NanoString Technologies (61). Patients with “hot” tumors were randomized to three cycles of durvalumab 1500 mg plus tremelimumab 75 mg every 4 weeks or standard cisplatin-based NAC, while patients in the “cold” arm received cisplatin-based NAC. In the “hot” arm, pCR was recorded in 36.4% of patients treated with NAC and in 34.8% of patients receiving durvalumab/tremelimumab. In the “cold” arm, as many as 68.8% of patients achieved pCR. Grade 3-4 toxicities occurred more frequently in the NAC group.

### ICIs and Chemotherapy

Conventional chemotherapy can elicit a tumor-specific immune response by inducing immunogenic cell death of neoplastic cells or engaging immune effector mechanisms (70). The combination of chemotherapy with immunotherapy has been extensively investigated. A phase II, single-arm trial, BLASST-1, examined the efficacy and safety of neoadjuvant nivolumab with GC for MIBC (cT2-T4aN ≤ 1M0) (62). Patients received four cycles of GC with nivolumab every 21 days, followed by RC within 8 weeks. Pathologic response (≤pT1N0) was observed in 65.8% of patients, including those presenting N1 disease. Safety profile was favorable, with 20% of grade 3-4 AEs mainly due to GC.

In patients with operable MIBC (cT2-4aN0-1), the open-label single-arm phase II trial, SAKK 06/17, tested neoadjuvant durvalumab plus GC (4 cycles every 21 days) followed by durvalumab monotherapy (10 cycles every 28 days) after surgery. Pathologic response was observed in 60% of patients, with 18 (34%) achieving pCR. Treatment demonstrated acceptable safety, and data regarding the primary endpoint, i.e. event-free survival (EFS) at 2 years, are awaited (71). Another multicenter, single-arm phase II trial enrolled eligible patients with MIBC (cT2-4aN0M0) to receive a dose of atezolizumab, followed 2 weeks later by GC plus atezolizumab every 21 days for 4 cycles, and after a further 3 weeks by a dose of atezolizumab prior to RC. The primary endpoint, downstaging to < pT2N0, was met in 27 (69%) patients including 16 (41%) pT0N0, all of whom experienced improved relapse-free survival. Grade 3 iAEs occurred in 5 (11%) patients with 2 (5%) requiring systemic steroids (72).

Efficacy and tolerability of neoadjuvant pembrolizumab and GC were assessed in a phase I/II trial, HCRN GU14-188, where patients with MIBC (cT2-4aN0M0) were subdivided into two cohorts: cisplatin-eligible (cohort 1) and cisplatin-ineligible (cohort 2) (63, 64). In cohort 1, pathologic response (≤pT1N0) and pCR were seen in 61.1% and 44.4% of patients, respectively. Median time from last dose to RC was 5.3 weeks; the 36-month relapse-free survival and OS were 63% and 82%, respectively. One death from mesenteric ischemia was recorded.

Phase III trials of neoadjuvant immunotherapy, comprising nivolumab, pembrolizumab and toripalimab, in combination with cisplatin-based chemotherapy are ongoing, and results are eagerly awaited (NCT03732677, NCT03661320, NCT03924856, NCT04861584). Several trials are also evaluating immunotherapy with non-cisplatin-based chemotherapy, including nab-paclitaxel and gemcitabine as neoadjuvant treatment. Among these, tislelizumab (BGB-A317), a humanized monoclonal antibody against PD-1, is being tested with nab-paclitaxel in MIBC (NCT04730219) (**Table 4**).

**Table 4:** Recruiting or active, not recruiting phase II and III clinical trials with neoadjuvant therapy for MIBC.

**Trial**	**Status**	**Phase**	**N of Patients**	**Neoadjuvant Treatment**	**Primary endpoint**
**NCT04861584**	Recruiting	II/III	41	Toripalimab with gemcitabine and cisplatin	Pathologic RR evaluated after 4 cycles
**NCT04060459**	Recruiting	II	50	Paclitaxel-binding albumin + cisplatin	pCR (<pT0)
**NCT03472274**	Recruiting	II	99	Durvalumab + tremelimumab	Antitumor activity
**NCT03674424**	Recruiting	II/III	166	Avelumab± chemotherapy	pCR (ypT0/TisN0)
**NCT04700124**	Recruiting	III	784	Perioperative enfortumab vedotin plus pembrolizumab vs. NAC	pCR EFS
**NCT04730219**	Recruiting	II	69	Tislelizumab with nab-paclitaxel	pCR
**NCT04543110**	Recruiting	II	25	Radiation + durvalumab	pCR
**NCT02690558**	Active, not recruiting	II	39	Pembrolizumab with gemcitabine and cisplatin	Pathologic downstaging
**NCT03732677**	Active, not recruiting	III	988	Durvalumab + gemcitabine/cisplatin (neoadjuvant treatment) and durvalumab (adjuvant treatment)	pCR EFS
**NCT04209114**	Recruiting	III	540	Nivolumab plus bempegaldesleukin vs. nivolumab alone vs. standard of care cisplatin ineligible	pCR EFS
**NCT02736266**	Recruiting	II	90	Pembrolizumab	pCR
**NCT04430036**	Recruiting	II	42	AGEN1884 +AGEN2034 with cisplatin-gemcitabine	Pathologic tumor downstaging of >T2 to pT0
**NCT03924856**	Recruiting	III	870	Perioperative pembrolizumab + NAC vs. perioperative placebo + NAC	pCR EFS
**NCT03294304**	Active, not recruiting	II	43	Nivolumab + gemcitabine and cisplatin	PaR
**NCT03558087**	Active, not recruiting	II	76	Gemcitabine and cisplatin + nivolumab	Clinical CR rate (cT0-Ta)
**NCT04289779**	Recruiting	II	42	Cabozantinib with atezolizumab	pRR
**NCT04047693**	Recruiting	II	32	Dose dense MVAC in MIBC and locally advanced urothelial carcinoma	pCR
**NCT02365766**	Active, not recruiting	I/II	83	Neoadjuvant pembrolizumab with gemcitabine	Rate of pathologic muscle invasive response
**NCT04383743**	Recruiting	II	17	Pembrolizumab and chemotherapy	pCR
**NCT02451423**	Recruiting	II	42	Atezolizumab	Change in CD3+ T cell count/µm2 in multi-dose cohorts; pathologic T0 rate in expansion cohorts
**NCT03061630**	Recruiting	II	48	Chemotherapy with gemcitabine/platinum	PaR
**NCT03768570**	Recruiting	II	238	Trimodality therapy with/out durvalumab	DFS
**NCT00777491**	Active, not recruiting	II	70	Chemotherapy and radiation therapy in stage II-III BC	Percentage of patients without distant metastases at 3 years
**NCT02845323**	Recruiting	II	44	Nivolumab ± urelumab in cisplatin-ineligible or chemotherapy-refusing patients	Immune response measured by tumor infiltrating CD8+ T cell density at cystectomy

Muscle invasive bladder cancer (MIBC), number (N), response rate (RR), pathologic complete response (pCR), event-free survival (EFS), pathologic response (PaR), disease-free survival (DFS), methotrexate-vinblastine-doxorubicin-cisplatin (MVAC).

### ICIs and Antibody-Drug Conjugates

Antibody-drug conjugates (ADCs), i.e. enfortumab vedotin and sacituzumab govitecan, are complex engineered therapeutics consisting of monoclonal antibodies directed toward tumor-associated antigens, to which highly potent cytotoxic agents are attached by chemical linkers (73). Enfortumab vedotin, a fully human monoclonal antibody conjugated to a clinically validated microtubule-disrupting agent, has shown encouraging results. Accordingly, the FDA has granted its accelerated approval in patients with locally advanced or metastatic urothelial carcinoma, formerly treated with PD-1/PD-L1 inhibitors and platinum-containing chemotherapy (74). At the 2022 American Society of Clinical Oncology (ASCO) meeting, Petrylak et al. presented the EV-103 phase Ib/II study evaluating antitumor activity of neoadjuvant treatment with enfortumab vedotin monotherapy in cisplatin-ineligible MIBC patients (75). Two randomized phase III trials are currently comparing perioperative enfortumab vedotin plus pembrolizumab with chemotherapy in cisplatin-eligible patients (NCT04700124) and with cystectomy alone in cisplatin-ineligible patients (NCT03924895). Sacituzumab govitecan is a humanized anti-trophoblast surface antigen 2 (Trop-2) antibody conjugated with SN-38, the active metabolite of irinotecan (76). The FDA has recently approved sacituzumab govitecan for patients with locally advanced or metastatic BC, previously administered platinum-based chemotherapy and PD-1 or PD-L1 inhibitors. At the 2021 GU ASCO Annual Meeting, Necchi et al. presented the design for the SURE trial assessing the efficacy of neoadjuvant sacituzumab govitecan, either as a single-agent (SURE-01) or combined with pembrolizumab (SURE-02), prior to RC in MIBC patients unfit for or refusing cisplatin-based chemotherapy (77).

### ICIs and Emerging Agents

A single-arm, phase II trial (NEODURVARIB) explored the impact of neoadjuvant durvalumab plus olaparib, a poly ADP-ribose polymerase inhibitor, in cT2-4N0 urothelial carcinoma (78). Patients received durvalumab 1500 mg every 4 weeks for a 2-month maximum (up to 2 doses/cycle) plus olaparib 300 mg for up to 56 days (2 cycles of 28 days each cycle). The pCR rate was 44.5% and grade 3-4 AEs occurred in 8.3% of patients, with one death related to postoperative complications. In the ongoing ABATE trial, the efficacy and safety of cabozantinib (a multikinase inhibitor of c-MET, AXL and VEGFR2) plus atezolizumab is being tested as neoadjuvant therapy for cT2-T4N0M0 BC patients who are either ineligible for or decline cisplatin (79).

## Predictive Biomarkers of Immunotherapy Response

With the emergence of immunotherapy, attempts have been made to identify biomarkers to predict clinical response. To date, potential biomarkers such as PD-L1 expression, CD8+ T-cell infiltration, DDR gene alterations, tumor mutational burden (TMB) and immune and stromal gene expression signatures have been correlated with immunotherapy response (80, 81). Nevertheless, none of these markers has shown consistent findings to warrant incorporation into BC routine management.

Controversial results on the role of PD-L1 as a predictive biomarker have been reported (82). PD-L1 positivity, detected in 20-30% of bladder tumors, appears to correlate with more advanced disease and poor prognosis (83). However, PD-L1 expression may depend on biopsy site and previous treatments, but primarily on the assays to test PD-L1 status (i.e. Dako 22C3, Ventana SP142, and Dako 28.8) based on the different ICI used. It should therefore be interpreted in the context of a broader biomarker panel, including neutrophil/lymphocyte ratio, albumin levels, high C-reactive protein and interleukin-6 (IL-6) levels. These can be easily integrated into clinical practice, unlike other more complex biomarkers such as gene expression signatures (84).

Investigation into the DDR pathways could be valuable for establishing the potential utility of immunotherapy. In a study analyzing a panel of 34 DDR genes, patients with advanced BC and deleterious mutations in these genes significantly benefitted from immunotherapy compared to those without DDR alterations (85). Moreover, ICIs have recently been reported to be highly effective in tumors with defects in the MMR/microsatellite instability pathway (86).

In the early phases of BC, the integrity of the immune system seems to induce a greater T-cell expansion than in advanced stages, characterized by increased impairment of T-cell function and cancer-associated inflammation. As a consequence, immunotherapy efficacy with checkpoint inhibitors has been explored in early-stage disease (87).

Recently, Mariathasan et al. demonstrated that inflamed and desert immune phenotypes were associated with response and resistance to atezolizumab, respectively (88). In this study, CD8 levels were higher in responding tumors, while elevated levels of fibroblast activation protein (FAP) were linked to immunotherapy resistance. In the PURE-01 study, PD-L1 expression, TMB, DDR and RB1 gene alterations were significantly related to pCR (57). Conversely, in the ABACUS trial, pCR correlated with granzyme B (GZMB) expression, a surrogate marker of activated CD8+ T cells (59).

Since several biomarkers, such as TMB and DDR alterations, are associated with the efficacy of both chemotherapy and immunotherapy, it may be difficult to select cisplatin-eligible patients and decide upon integration and sequencing of different therapeutic options in the multimodal management of MIBC (89, 90). Notably, cytotoxicity induced by NAC can elicit an immune effect by activating CD8+ T cells and decreasing Tregs (91). This would impede T-cell response when NAC and immunotherapy are administered concurrently, and partly explain the limited benefit of NAC plus immunotherapy compared with NAC alone in advanced urothelial cancer (92). NAC followed by immunotherapy could therefore be a more effective approach.

## Potential Neoadjuvant Agents in MIBC

**Figure 1:**
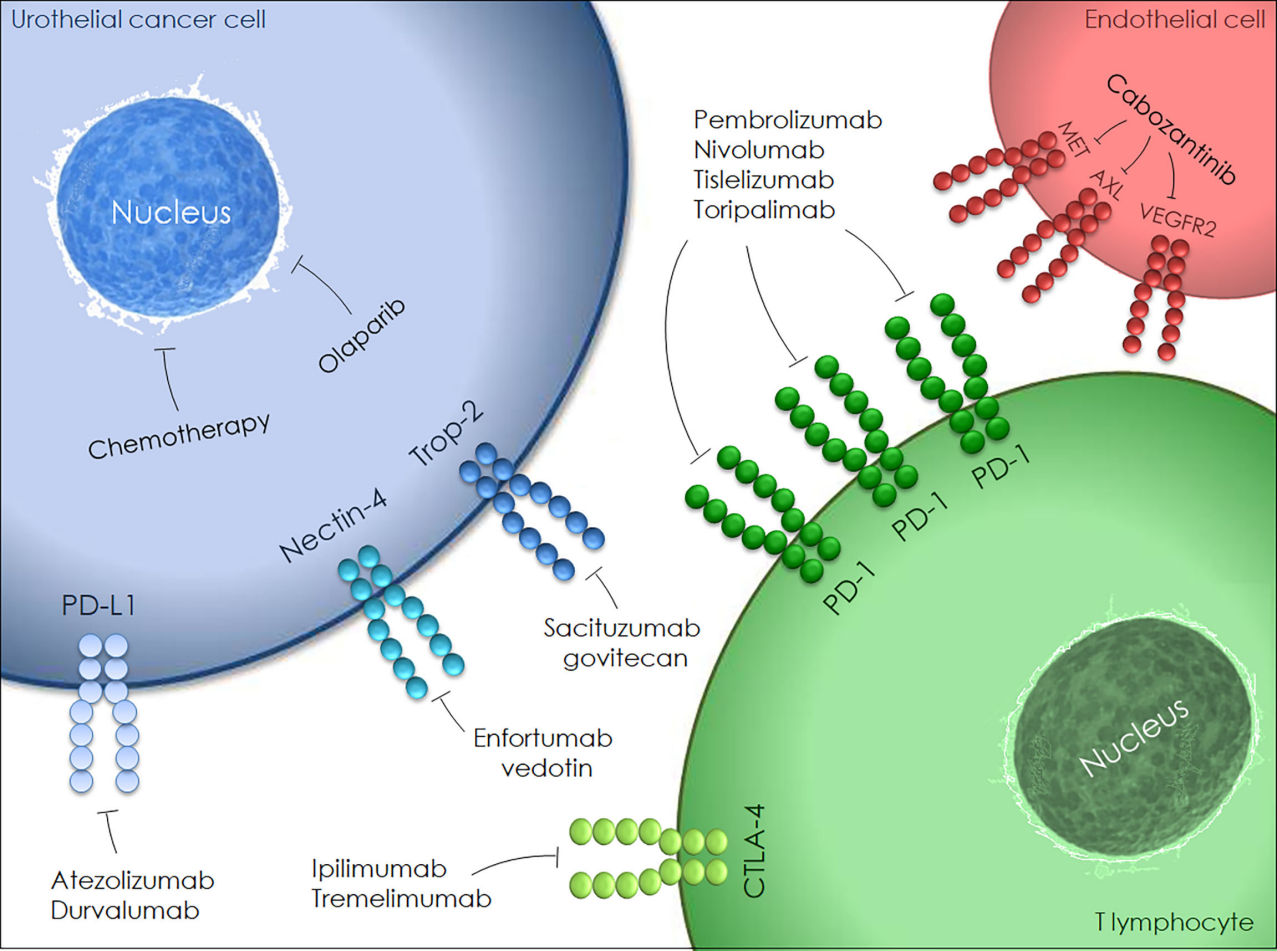
Emerging agents in the context of neoadjuvant setting for patients with MIBC.

Emerging neoadjuvant agents are illustrated in **Figure 1**. The genetic alteration of the fibroblast growth factor receptor (FGFR) pathway has been widely investigated in BC with subsequent approval of FGFR inhibitors in advanced and metastatic settings. Infigratinib (FGFR1-3-selective tyrosine kinase inhibitor) monotherapy is currently being tested as neoadjuvant treatment for locally advanced urothelial cancer (NCT0422804).

Bempegaldesleukin (BEMPEG/NKTR-214) is a PEGylated interleukin-2 (IL-2) designed to activate and proliferate CD8+ T cells and natural killer (NK) cells. An in-progress randomized study is comparing BEMPEG plus nivolumab with nivolumab alone for neoadjuvant and adjuvant treatment of cisplatin-ineligible resectable MIBC patients (NCT04209114).

Urelumab is a fully human IgG4 monoclonal antibody that targets the CD137 extracellular domain stimulating cytotoxic T cell responses against tumor cells. A trial assessing the efficacy of nivolumab monotherapy or combined with urelumab in cisplatin-ineligible or chemotherapy-refusing MIBC patients is ongoing (NCT02845323). Further trials are assessing novel agents, namely CD73 inhibitor (NCT03773666), replication-competent oncolytic adenovirus (NCT04610671) and synthetic benzamide-derivative histone deacetylase inhibitor (NCT03978624).

## Radiotherapy in MIBC

Neoadjuvant radiation should not be used in patients with MIBC prior to RC. Although preoperative radiotherapy, as a single modality, can eradicate disease in a small proportion of patients undergoing cystectomy, it seems to improve local control rather than survival when compared with RC alone (93). However, radiation can synergize with immunotherapy to improve clinical outcomes by causing immunogenic cell death and increasing expression of immune markers (94). Following this hypothesis, several trials, such as RADIANT (durvalumab + radiotherapy) (NCT04543110) and RACE IT (nivolumab + radiotherapy) (NCT03529890) prior to cystectomy in MIBC, are still active. The efficacy of chemotherapy and radiation therapy in stage II and III bladder carcinoma patients is also being tested (NCT00777491).

## Bladder Cancer Molecular Subtypes and Therapeutic Implications

**Figure 2:**
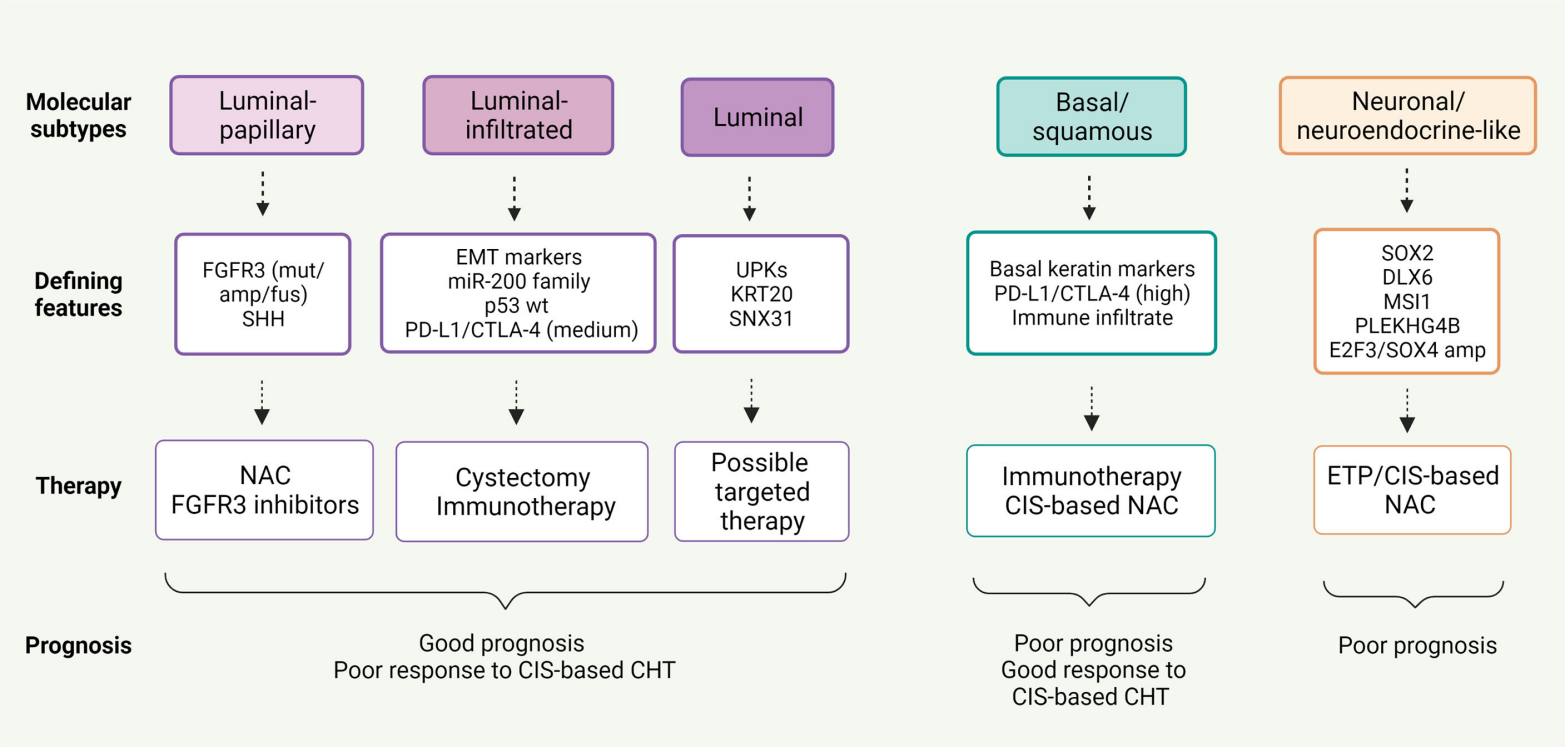
Correlation between five mRNA-based expression subtypes according to The Cancer Genome Atlas (TCGA) analysis and response to neoadjuvant therapy in MIBC.

Potential markers and gene expression models have been correlated to chemotherapy response in BC (32, 95, 96), but none have been approved for clinical practice as yet. However, new insights into BC molecular pathology could lead to a shift toward individualized treatment and consequently better patient outcomes (**Table 5** and **Figure 2**).

**Table 5:** Subtypes of bladder carcinoma according to different molecular classifications.

**Classification**	**N of patients**	**Patients**	**Subtypes**	**Molecular characteristics**
**Lund University (2012)** (97)	308	BC	Urobasal A	High FGFR3, CCND1 and P63 expression
			Urobasal B	
			Genomically unstable	TP53 mutations; high CCNE and ERBB2 expression; low cytokeratin expression
			Squamous cell carcinoma-like	High expression of basal keratins
			Infiltrated	Stromal and immune cell infiltration
**UNC (2014)** (98)	262	High grade MIBC	Luminal	Expression of E-cadherin/CDH1 and miR-200; FGFR3 alterations
			Basal	High EGFR expression
**MDA (2014)** (99)	73	MIBC	Luminal	FGFR3 mutations
			Basal	P63 activation
			P53-like	P53 signature activation
**TCGA (2012)** (97)	131	High grade MIBC	Cluster I	Luminal phenotype
			Cluster II	Luminal phenotype with P53-like features
			Cluster III	Corresponding to basal subtype of UNC and MD Anderson classifications
			Cluster IV	
**TCGA (2017)** (100)	412	MIBC (T2-4, N0-3, M0-1)	Luminal-papillary	FOXA1, GATA3 and PPARG expression, FGFR3 alterations
			Luminal-infiltrated	Expression of FOXA1, GATA3, PPARG, EMT and immune markers
			Luminal	Expression of FOXA1, GATA3, PPARG, KRT20
			Basal/squamous	CD44 and KRT5/6 expression; TP53 mutations
			Neuronal	Neuroendocrine and neuronal marker expression
**BCMTG (2020)** (101)	1750	MIBC	Luminal-papillary	FGFR3 and PPARG expression; FGFR3, ELF3 and KDM6A mutations
			Luminal non-specified	PPARG, E2F3 and ERBB2 expression; TP53 and ERCC2 mutations
			Luminal unstable	EGFR expression; TP53 and RB1 mutations
			Stroma-rich	Neuroendocrine differentiation; loss or mutations of TP53 and RB1

Muscle invasive bladder cancer (MIBC), bladder cancer (BC), number (N), University of North Carolina (UNC), MD Anderson (MDA), The Cancer Genome Atlas (TCGA) (Bladder Cancer Molecular Taxonomy Group (BCMTG).

Basal/squamous tumors, defined by the Cancer Genome Atlas (TCGA), University of North Carolina (UNC) and MD Anderson classifications (98, 99, 102), are associated with more advanced stages and worse prognosis, whereas luminal tumors appear to be less aggressive (37, 100). Patients with basal tumors seem to profit more from NAC than those with luminal tumors who derive little or no benefit. Irrespective of treatment strategy, luminal-papillary tumors bear the best prognosis, unlike luminal-infiltrated tumors that have an unfavorable prognosis regardless of NAC (103, 104).

Furthermore, these molecular classifications can balance standard histologic classifications burdened by intra- and intertumoral heterogeneity of primary MIBC, with relevant clinical implications. Compared with transcriptome analysis, immunohistochemistry (IHC) is a simpler and more accessible method to classify urothelial carcinoma into molecular subtypes, comprising basal (KRT5/6, KRT14, and p63) and luminal (GATA3, FOXA1, uroplakins and HER2) phenotypes.

To stratify BC patients, Makboul et al. utilized a simple IHC panel of five biomarkers, i.e. FGFR3, KRT5, cyclin B1, HER2 and p53 (105). The molecular classes identified were correlated with clinico-pathologic variables and patient survival: basal/squamous tumors showed the lowest OS (38.5%), while urobasal A (UroA) tumors, expressing luminal markers, had the best prognosis with OS of 75% and no metastatic events. In addition, Choi et al. found that basal tumors had a significantly higher response rate to cisplatin-based chemotherapy, and all chemoresistant tumors exhibited a p53-like phenotype (99).

A recent study by Font et al. stratified MIBC patients receiving NAC into three subgroups, i.e. basal/squamous (KRT5/6 and KRT14 high; FOXA1 and GATA3 low), luminal (FOXA1 and GATA3 high; KRT5/6 and KRT14 low) and mixed (FOXA1 and GATA3 high; KRT5/6 high and KRT14 low), using IHC combined with hierarchical clustering analysis. Overall, pathologic response to NAC was significantly higher in patients with basal/squamous tumors (p=0.017) (106).

Through the use of transcriptome-wide gene expression and IHC, Seiler et al. categorized the residual tumor at cystectomy after NAC, and outlined the greatest potential benefit from second-line treatments, such as checkpoint inhibition, in tumors with high immune infiltration, elevated expression of immune-associated genes (i.e. CTLA4, MPEG1 and CD27) and low expression of basal or luminal markers (107).

Likewise, correlation between tumor subtypes and efficacy of immunotherapy has recently been explored. The revised TCGA classification suggested that patients with luminal-infiltrated tumors benefit most from immunotherapy (100). In the IMvigor 210 study, treatment with atezolizumab was most beneficial in advanced BC classified as TCGA cluster II (108), whereas basal tumors were more likely to respond to nivolumab in the CheckMate 275 study (49). Despite the high immune infiltration, response to immunotherapy was poor in claudin-low tumors, defined by biologic characteristics of the claudin-low subtype of breast cancer, probably due to more effective T cell suppression in cluster IV than cluster II tumors (109). IMvigor 210 trial also showed that survival advantage of atezolizumab was greater in TCGA neuronal-subtype tumors, without this being related to other predictors of immunotherapy response, such as TMB and tumor neo-antigen load (110).

Molecular classification of BC according to gene expression profiles can play a crucial role in determining the most suitable treatment. Immunotherapy and chemotherapy appear to be advantageous in complementary patient populations. Patients with luminal tumors show better prognosis but poor response to cisplatin-based chemotherapy. Cystectomy is the best option in these patients, however, immunotherapy may be beneficial in luminal-infiltrated tumors. On the contrary, chemotherapy proves to be the treatment of choice in basal tumors.

## Conclusions

Despite guideline recommendations, NAC prior to cystectomy is still seldom adopted. Newly developed therapies, such as immunotherapy, targeted therapy and combination strategies, are being tested in clinical trials. The use of biomarkers to predict response to cisplatin-based NAC or ICIs is largely investigational, but molecular signatures are showing promise in reshaping selection for tailored treatment and disease monitoring.

## Author Contributions

Study concepts: GR, GN. Study design: GR, MC. Data acquisition: RS, MS, IG. Quality control of data and algorithms: UG. Manuscript preparation: MC, GR, GN. Manuscript editing: MS, WP, AA. Manuscript review: UG, AA. All authors contributed to the article and approved the submitted version.

## Conflict of Interest

UG received honoraria for advisory boards or invited speaker fees from Pfizer, BMS, MSD, PharmaMar, Astellas, Bayer, Ipsen, Novartis, Roche, Clovis, AstraZeneca, institutional research grants from AstraZeneca, Sanofi and Roche.

The remaining authors declare that the research was conducted in the absence of any commercial or financial relationships that could be constructed as a potential conflict of interest.

## Publisher’s Note

All claims expressed in this article are solely those of the authors and do not necessarily represent those of their affiliated organizations, or those of the publisher, the editors and the reviewers. Any product that may be evaluated in this article, or claim that may be made by its manufacturer, is not guaranteed or endorsed by the publisher.
